# Expert consensus-based recommendations on the use of photodynamic therapy in actinic keratosis patients

**DOI:** 10.1016/j.jdin.2024.11.011

**Published:** 2025-03-13

**Authors:** Vishal A. Patel, Sarah T. Arron, Brian Berman, M. Shane Chapman, Anokhi Jambusaria-Pahlajani, George Martin, Anthony M. Rossi, Todd Schlesinger, Nathalie C. Zeitouni, Neal Bhatia

**Affiliations:** a Department of Dermatology, George Washington University School of Medicine & Health Sciences, Washington, District of Columbia; b Peninsula Dermatology, Burlingame, California; c University of Miami Miller School of Medicine, Miami, Florida; d Center for Clinical and Cosmetic Research, Aventura, Florida; e Department of Dermatology, Dartmouth Hitchcock Medical Center, Nashua, New Hampshire; f Geisel School of Medicine at Dartmouth, Lebanon and Hanover, New Hampshire; g Division of Dermatology, Department of Internal Medicine, The University of Texas at Austin Dell Medical School, Austin, Texas; h Dr. George Martin Dermatology Associates, Kihei, Hawaii; i Memorial Sloan Kettering Cancer Center, New York, New York; j Weill Cornell Medical College, New York, New York; k New York Presbyterian Hospital, New York, New York; l Clinical Research Center of the Carolinas, Charleston, South Carolina; m Medical Dermatology Specialists, Phoenix, Arizona; n University of Arizona COM, Phoenix, Arizona; o Therapeutics Clinical Research, San Diego, California

**Keywords:** actinic keratosis, aminolevulinic acid (ALA), basal cell carcinoma (BCC), cutaneous squamous cell carcinoma (cSCC), field therapy, organ transplant recipients, photodynamic therapy, red light

## Abstract

**Background:**

Current actinic keratosis (AK) treatment guidelines are controversial and incomplete, leaving an unmet need for clearer and more in-depth guidance on the usage of photodynamic therapy (PDT) in AKs.

**Objective:**

To gain consensus on the use of PDT for AKs using a modified Delphi method.

**Methods:**

Consensus statements were developed after a literature review of topics surrounding PDT and AKs. Ten panelists voted through electronic questionnaires rating each statement on a three-point Likert-type scale: strongly agree, agree, and disagree. Consensus was established when ≥70% of panelists strongly agreed on the statement. Statements that did not meet the threshold were revised after each round of voting and sent out for the next round of voting. A virtual meeting was held after 2 rounds of voting to discuss outstanding statements.

**Results:**

Three modified Delphi rounds were completed in total and 1 virtual meeting was held. All 10 experts responded to all rounds of voting. A total of 55 statements established consensus with the ≥70% majority requirement necessary.

**Limitations:**

Recommendations are based on expert opinions.

**Conclusion:**

The results from this consensus panel are intended to bridge gaps in the existing guidelines and will continue to evolve.

**Capsule Summary**

• There is a need for clearer and in-depth guidance in the usage of photodynamic therapy for actinic keratoses.
• This modified Delphi method consensus group has established 55 statements surrounding the use of photodynamic therapy in treating actinic keratoses to bridge gaps in existing guidelines.

## Introduction

Actinic keratoses (AKs) are premalignant skin lesions that occur on sun-exposed skin in up to 26% of Americans over 30 years of age.^1^, ^2^, ^3^ Cutaneous squamous cell carcinomas (cSCCs) can progress from AKs, and individuals found with more than 5 AKs are more at risk.^4^ The estimated rate of conversion from AK to cSCC is approximately 10% with a 2-year progression timeline.^5^ Over 40% of patients with a prior diagnosis of multiple AKs developed nonmelanoma skin cancers after 5-11 years.^6^^,^^7^ Studies have shown that previously treated area of AKs have a 3.7% total 4-year risk of developing cSCC and increases to 20.9% in those with severe AKs.^8^ Patients with immune dysfunction, such as solid-organ transplant recipients (SOTRs), have an increased risk of developing cSCCs due to their immunocompromised status as well as increased actinic burden.^6^^,^^9^, ^10^, ^11^, ^12^

Several treatment options are currently available for AK treatment including lesion-directed options such as surgery and cryotherapy, and field-directed options such as laser treatments, chemical peels, topical therapies, and photodynamic therapy (PDT).^13^ According to the 2021 American Academy of Dermatology guidelines, the current level of recommendation for use of topicals such as 5-fluorouracil (5-FU), imiquimod, and tirbanibulin cream is strong, with moderate quality of evidence. Cryosurgery also has a strong level of recommendation.^14^ Diclofenac and combinational therapy carry a conditional level of recommendation, with between low and moderate quality of evidence.^14^ But most surprising is that PDT also has a conditional level of recommendation, with reported low to moderate quality of evidence, despite several trials as well as its approval for the treatment of AK.^14^

PDT is a noninvasive treatment option for AKs that can be used to treat large areas of field cancerization.^15^, ^16^, ^17^, ^18^ Although PDT is a newer procedure, it has proven safety and efficacy in treating AKs.^16^, ^17^, ^18^ It can improve patient compliance as an in-office procedure, while also resulting in improved cosmetic outcomes.^14^ PDT has been reported to be well-tolerated by patients.^19^^,^^20^ In a prospective comparative study, patients reported no significant difference in tolerability between PDT and other field treatments.^19^ In another randomized trial of 4 field treatments for AKs, similar percentages of adverse events were reported in PDT, ingenol mebutate, and 5-FU, while imiquimod had the least.^20^ In a treatment preference study, patients reported equal preference for cryotherapy, trichloroacetic acid destruction, and PDT and preferred PDT over the topical treatments imiquimod and 5-FU in the treatment of AKs.^21^ Patients undergoing traditional PDT may experience pain; however, newer PDT protocols have been developed to address pain levels as well as convenience.^22^

Other procedural field treatments such as lasers and chemical peels have been used in the treatment of AKs.^23^ Although chemical peels are a cost-effective, well-tolerated, one-off treatment, they are significantly less effective at treating AKs than PDT and carry the risk of scarring and infection.^23^, ^24^, ^25^ Lasers have also seen success in treating AKs, however, it is not a targeted treatment for AK but can provide improvement in some lesions as a result of skin rejuvenation.^26^ In addition, it is a more costly treatment option and is not covered by insurance companies. The American Academy of Dermatology guidelines conditionally recommend cryosurgery over laser ablation with moderate quality evidence.^14^

Current AK guidelines are both controversial and incomplete, therefore there is a need for clear, more in-depth recommendations for PDT.^14^ Our goal was to gain consensus on the use of PDT for AKs using a modified Delphi method with a panel of 10 subject matter experts.^26^^,^^27^ Topics included the role of PDT in field cancerization, cancer prevention with PDT, when to change from spot treatment to field treatment, ideal patient types, compliance, patient preferences, cosmetic outcomes, and general PDT usage. This article reports the development and recommendations of the Photodynamic therapy in Actinic Keratosis Treatment (PAKT) Consensus Group.

## Methods

A systematic literature review surrounding topics within PDT for AKs was conducted. The purpose of this search was to review existing evidence to aid in consensus statement development. The PubMed database was queried for all relevant journal articles from 1990 to 2023 using exploded Medical Subject Headings terms and keywords in the following topics: patient types, compliance, adherence, pain, alternate treatment options (ie, radiotherapy, cryosurgery, topicals), squamous cell carcinoma, field treatment, light source, incubation times, spot treatment, and patient preference. The term “AND” was used to find relevant papers with those topics in the field of “actinic keratosis” and “photodynamic therapy.” All randomized controlled trials, prospective and retrospective studies, case studies, peer-reviewed articles, major international conference abstracts, narrative reviews, and editorials written in English were included.

Professional medical writers independently reviewed the literature and developed consensus statements. Ten panelists of dermatologists were selected for their expertise in treating AKs and use of PDT. Consensus was sought through voting rounds sent as an electronic questionnaire using a modified Delphi method. Panelists were asked to rate each statement on a three-point Likert-type scale: disagree, agree, and strongly agree. Panelists were required to make recommendations on statement modification and to provide the source for their recommendation if they selected agree or disagree on each question, the answers were then used to refine statements. To establish consensus, ≥70% of panelists must strongly agree with the statement. If the 70% threshold was not met, the statement was revised according to panelist’s comments and put through the next round of voting. A virtual meeting was convened after 2 initial rounds of electronic voting to discuss statements that had not met consensus. Anonymity was maintained throughout the process to avoid bias. Panelists did not turn on their cameras and did not use their real names during the virtual meeting. Statements which could not be refined during the virtual meeting were removed completely. The entire process was run by an independent medical education group funded by an unrestricted educational grant and without influence from sponsoring entities.

## Results

Three Delphi rounds were completed in total. Ten dermatologists with expertise in dermatologic oncology were invited to participate in this consensus group. Of these, all 10 responded to all rounds, however, 1 panelist did not give an opinion on some of the statements during both the second and third round of voting. This did not affect the results as a minimum of 70% panelists were still required to strongly agree to reach consensus. A virtual meeting was convened in December 2023, and all panelists were there to discuss proposed consensus statements that required amending. The panelists considered and amended statements across 5 broad categories. The first category detailed recommendations for general aspects of PDT in AKs (Table I) which had a total of 16 statements that achieved ≥70% strong agreement. Other categories include patient selection and preference for PDT in AKs (Table II) with a total of 7 statements, other treatment options and sequential treatment for PDT in AKs (Table III) with 8 statements, PDT protocols for AKs (Table IV) with 13 statements, and PDT in AKs for special populations such as organ transplant recipients (OTRs) and the elderly (Table V) with 11 statements. In total, the consensus group yielded 55 statements that passed the ≥70% majority requirement necessary to be included in these treatment recommendations.

**Table I:** Consensus statements on general aspects of PDT in AKs

	Consensus *n* (%)
General PDT	
PDT should be considered a safe, efficacious, treatment modality for photodamaged fields and multiple AKs.^28^, ^29^, ^30^	10/10 (100%)
Addressing subclinical AKs in addition to evident AKs should result in longer remission.^31^ Furthermore, the goal of field treatment should be to delay or prevent the appearance of squamous cell carcinomas (cSCCS) and other cutaneous malignancies.^32^^,^^33^	8/10 (80%)
PDT is safe for all Fitzpatrick skin types.^34^	8/10 (80%)
PDT should be used as a strong therapeutic option for chemoprevention due to its ability to reduce the risk of cSCC and is currently underused in the US market.^35^^,^∗	7/10 (70%)
The experience of the consensus panel shows that field treatment of AKs does result in fewer future NMSC malignancies.^36^^,^^37^^,^∗	8/10 (80%)
PDT is an in-office procedure and generally covered by insurance, including Medicare. Topical treatments are covered by drug plans. Not all patients have drug plans, including Medicare patients who may have significant out-of-pocket expenses.∗	7/10 (70%)
For patients with nonhypertrophic AKs, PDT may be a good option due to short treatment duration and improved cosmetic outcomes^.^∗	9/10 (90%)
PDT may be used as part of a regimen for chemoprevention of cutaneous SCCs in organ transplant recipients.^38^	10/10 (100%)
Cyclic PDT performed every 2 mo may be considered to suppress the development of new cSCCs in sOTRs.^36^	10/10 (100%)
Lesion vs field-directed therapy	
Because we are uncertain which AK lesions will likely progress to cSCC, all lesions both clinical and subclinical need to be treated.^32^^,^^39^^,^∗	10/10 (100%)
Field-directed PDT yields favorable outcomes (cancer prophylaxis and cosmetic) in patients with high levels as well as low levels of actinic damage.∗	8/10 (80%)
Spot treatment used to treat isolated, clinically evident AK lesions can be optimized when followed by field treatment to treat both evident and subclinical lesions.^18^^,^^32^^,^^39^	7/10 (70%)
Cosmetic outcomes	
Field-directed PDT is associated with improvements in roughness, dryness, and scaling and decreases in hypopigmentation and hyperpigmentation of the treatment area with a lower potential of scarring.^15^^,^^40^^,^^41^	9/10 (90%)
Outside of contraindications, PDT should be part of a standard regimen to reduce skin cancer risks, especially where minimal downtime, esthetic impact, and economic impact are considerations.∗	8/10 (80%)
PDT has a rejuvenation effect on the skin, often resulting in a reduction in the appearance of photodamage including reduced lentigines, skin smoothness, brightness, and a more even tone.^42^^,^∗	7/9 (77.8%)
For better cosmetic outcomes, combinations with other agents should be incorporated into a long-term PDT regimen, eg, retinoids, photolyases, sunscreens, and nicotinamides.∗	7/10 (70%)

*AK*, Actinic keratosis; *cSCC*, cutaneous squamous cell carcinoma; *NMSC*, nonmelanoma skin cancer; *PDT*, photodynamic therapy; *sOTR*, solid organ transplant recipient.

∗ The consensus statement was based on the collective experience of the panelists.

**Table II:** Consensus statements on patient selection and preference for PDT in AKs

Patient selection	Consensus *n* (%)
Patients who may benefit the most from field therapy vs spot therapy are or have: • Multiple actinic keratoses (AKs), either on the face/scalp or extremities • Large areas of photodamage, either on the face/scalp or extremities • Patients who are on the spectrum of relative immunosuppression, including organ transplant recipients and those with diabetes, chronic lymphocytic leukemia and other iatrogenic sources. • A previous history of skin cancer • Refractory or noncompliant with other treatment options	8/10 (80%)
Patients who are/have the following are good candidates for field-directed PDT^33^^,^^43^, ^44^, ^45^, ^46^: • Lesions anywhere on the body • Undergone an organ transplant • NMSC or cSCC • Managing background actinic changes • A need for a maintenance treatment for low-grade AKs in sun-damaged skin • Any Fitzpatrick skin type • Refractive to other treatments • Any age, gender, race • History of AKs or recurring AKs	7/10 (70%)
Factors to be considered when selecting PDT as a treatment include age, gender, amount of actinic damage and pigmentary changes, level of hypertrophic AKs, surface area to be treated, Fitzpatrick skin type, and tolerability of a patient.∗	7/10 (70%)
PDT should be considered in patients with a history of multiple (>10) cSCCs and/or BCCs, including the face, arms, and legs.∗	7/10 (70%)
Patient preference	
Patients prefer treatments that have a minimal impact on daily life, simple regimen, short treatment duration, and low frequency of treatments.^47^, ^48^, ^49^, ^50^, ^51^	8/10 (80%)
PDT has a high adherence rate among patients, possibly because it is administered in an office setting.^41^^,^^52^^,^^53^	9/10 (90%)
Patients prefer treatments that do not cause scarring.^54^	10/10 (100%)

*AK*, Actinic keratosis; *BCC*, basal cell carcinoma; *cSCC*, cutaneous squamous cell carcinoma; *NMSC*, nonmelanoma skin cancer; *PDT*, photodynamic therapy.

∗ The consensus statement was based on the collective experience of the panelists.

**Table III:** Consensus statements on other treatment options and sequential treatment for PDT in AKs

Treatment options	Consensus *n* (%)
At-home topical AK treatments may have poor persistence and adherence when long treatment times and cosmetic downtime is involved.^20^	8/10 (80%)
Warming the skin with a heating pad, alpha hydroxy acid, or occlusion may improve AK reduction, but more clearly defined parameters should be outlined.^14^^,^^55^	8/10 (80%)
Sequential treatment and/or pretreatment options (5-FU, imiquimod, ablative lasers (ABL), microneedling) may yield better results than PDT alone.^56^, ^57^, ^58^, ^59^, ^60^	10/10 (100%)
Performing curettage on evident AK lesions enhances efficacy of field-directed therapies.^61^^,^∗	7/9 (77.8%)
Many small studies and case reports tout the beneficial effects of pretreatment with 5-FU, imiquimod, or AHA, prior to treatment with PDT.^55^, ^56^, ^57^, ^58^^,^^62^, ^63^, ^64^	8/10 (80%)
Sequential use of PDT and imiquimod is well tolerated and shown to improve efficacy in AK reduction.^58^^,^^65^	9/10 (90%)
Combination use of PDT and other field treatments can yield good efficacy.^55^, ^56^, ^57^, ^58^^,^^62^, ^63^, ^64^	7/10 (70%)
5FU possesses high efficacy compared to other field treatments but may not be favored by patients.^50^^,^^66^	9/10 (90%)

*5-FU*, 5-Fluorouracil; *ABL*, ablative lasers; *AHA*, alpha hydroxy acid; *AK*, actinic keratosis; *PDT*, photodynamic therapy.

∗ The consensus statement was based on the collective experience of the panelists.

**Table IV:** Consensus statements on PDT protocols for AKs

	Consensus *n* (%)
Photosensitizer	
The ALA nanoemulsion formulation can enhance the penetration of ALA through the skin as well as epidermal PpIX formation.^67^^,^^68^	9/10 (90%)
Both 10% ALA topical gel and 20% ALA topical solution are effective at clearing AK lesions with blue-light PDT, but the topical gel causes less local skin reactions.^69^	7/9 (77.8%)
ALA nanoemulsion gel is easier to use compared to an ALA topical solution due to reduced risk of unwanted running or migration of ALA during the application process.∗	7/9 (77.8%)
Pain during ALA-PDT is not related to Fitzpatrick skin type but may be related to AK clinical grade, treatment location, and incubation time if not using painless protocols.∗	8/10 (80%)
Incubation time	
Optimal incubation time is dependent on patient tolerance for pain and time commitment, ability for curettage on all hyperkeratotic AKs, and number, severity, and location of evident lesions.∗	8/10 (80%)
Outcomes may be enhanced when using shorter incubation times through the use of occlusion, higher temperatures in the room (eg, blankets or space heaters), curettage, and extra treatment sessions.^14^^,^^55^^,^∗	7/10 (70%)
Facial lesions are the most responsive to PDT and require the least incubation time, followed by the scalp and lastly, the extremities, which may require longer incubation times and/or adjuvant heat or occlusion.^53^^,^∗	7/10 (70%)
Light source	
Experts generally agree that red light penetrates deeper and may be better for chemoprevention but both red and blue light are effective for PDT treatment.^70^^,^∗	7/10 (70%)
Daylight PDT has been reported to be effective in some geographic locations and some providers have had success using it.^71^^,^^72^^,^∗	8/10 (80%)
Pulsed dye lasers and intense pulsed light are not efficient light sources for performing photodynamic therapy.^73^	9/10 (90%)
Painless PDT protocol	
Patients with low pain tolerance or time commitment can be treated effectively with PDT with the modified painless PDT protocol, but at least 2 treatment sessions are recommended.∗	9/10 (90%)
Results from the painless PDT protocol consisting of 0-30-minute incubation times can be enhanced with at least 2 treatment sessions and the addition of vitamin D3 twice a week.^74^^,^^75^^,^∗	8/10 (80%)
Patients may be more accepting and willing to undergo PDT when painless protocols are employed.∗	8/10 (80%)

*AK*, Actinic keratosis; *ALA*, aminolevulinic acid; *PDT*, photodynamic therapy; *PpIX*, protoporphyrin IX.

∗ The consensus statement was based on the collective experience of the panelists.

**Table V:** Consensus statements on PDT in AKs for special populations

	Consensus *n* (%)
Organ transplant recipients	
Conventional lesion-by-lesion-directed treatment of AKs may precede field therapy in some OTRs who have fewer lesions, but evaluation of the need for field therapy should be ongoing through regular follow-up to prevent progression to cSCC.^29^^,^^76^^,^∗	9/10 (90%)
Field-directed PDT yields favorable outcomes for nontransplant patients and organ transplant recipients.^46^^,^^77^, ^78^, ^79^, ^80^	8/10 (80%)
Chemopreventative field-directed PDT should be considered for OTR to mitigate AKs and reduce the risk of developing cSCCs.^79^	8/10 (80%)
Field treatment for AK is preferred compared to spot treatment for better disease control in organ transplant recipients.^43^	7/10 (70%)
Organ transplant recipients have good response rates to PDT.^78^^,^^79^^,^^81^^,^^82^	8/10 (80%)
Two sessions of PDT are more effective than 1 for AK clearance in organ transplant recipients.^28^	10/10 (100%)
Organ transplant recipients are highly satisfied with PDT, even with reporting higher pain levels, and prefer it to other treatments.^21^^,^^28^^,^^80^^,^^83^	7/10 (70%)
Pretreatment of AKs with curettage, keratolytics, lasers, microdermal abrasion, topical therapy, or cryotherapy can improve photosensitization for PDT in organ transplant recipients.^81^	9/10 (90%)
Elderly	
PDT should be considered for older patients due to the low incidence of ulceration and infection.^84^	8/10 (80%)
Treatments with high and sustained efficacy, like PDT, should be considered in older patients to reduce the risk of cSCC progression.^85^, ^86^, ^87^, ^88^, ^89^	7/10 (70%)
PDT may be preferable in older patients due to difficulty in managing topical applications, side effects, wound and infection risk, and dosing confusion. Furthermore, Medicare coverage may be better.^85^, ^86^, ^87^, ^88^, ^89^^,^∗	7/10 (70%)

*AK*, Actinic keratosis; *cSCC*, cutaneous squamous cell carcinoma; *OTR*, organ transplant recipient; *PDT*, photodynamic therapy.

∗ The consensus statement was based on the collective experience of the panelists.

## Discussion

The PAKT Consensus Group was able to provide recommendations regarding many facets of PDT in AK. In general, there was consensus that PDT should be utilized as a field treatment and as a means of chemoprevention due to its ability to treat both evident and subclinical AKs (Table I). The field treatment goal should be prevention or delaying the onset of nonmelanoma skin cancers and other malignancies, especially in vulnerable populations such as Gorlin’s syndrome or SOTRs.^90^ PDT is safe for all skin types; however, caution should be taken in darker skin types due to the risk of pigmentary damage, and shorter incubation times should be used initially.^91^ Panelists agreed that PDT is a strong therapeutic option but currently underused in the United States. Historical patterns of lesion-based destructive therapy are only beginning to be altered with a shift toward all types of field treatment. As the health macroeconomic and reimbursement landscapes change, field treatment proves to be a more cost-effective way to manage the growing epidemic on sun damage and skin cancer.

Our panelists developed clear criteria for types of patients who are good candidates and may benefit most from PDT (Table II). Panelists agreed that PDT should be considered in patients with a history of multiple (>10) cSCCs and/or basal cell carcinomas, including the face, arms, and legs, though other treatments should also be considered for use in conjunction to reduce field cancerization. Panelists noted that patients prefer treatments that have a minimal impact on daily life, simple regimen, short treatment duration, and low frequency of treatments.^47^, ^48^, ^49^, ^50^, ^51^ They also agreed that patient tolerability and comfort is an important consideration and encompasses pain, the need for temporary daylight avoidance, and cutaneous side effects. Overall, panelists agreed that PDT encompasses these criteria in many or most clinical scenarios and should be discussed as a treatment option for most patients. However, the clinician should consider the patient’s age, gender, patient medical history (ie, porphyria), amount of actinic damage and pigmentary changes, level of hypertrophic AKs, surface area to be treated, Fitzpatrick skin type and tolerability, when deciding if PDT may or may not be a good therapeutic option. Other factors to be considered include potential insurance coverage as well as patient preference and a family history of carcinoma cutis. The panel also identified that there is a need for dermatologists to educate patients about PDT benefits as a long-term strategy for field cancerization and photodamage and incorporate that into the treatment regimen.

When deciding whether to use lesion or field-directed therapy, the panelists recommended that PDT should be incorporated to optimize results from ongoing or previous spot treatment.^18^^,^^32^^,^^39^ Cycling topical treatment between PDT, while using sunscreen and photoprotective options, has also been suggested as an effective long-term strategy.^18^ Although PDT may yield favorable outcomes in both cancer prophylaxis and cosmesis in patients with both high and low levels of AK damage, patients with higher AK burdens may not clear despite multiple rounds of PDT, and thus may require the addition of a topical regimen.^92^ There was a strong consensus that PDT improves cosmetic outcomes as well as having a rejuvenation effect on the skin and should be considered when counseling patients and developing a treatment plan.^42^ Since AKs occur primarily in visible locations (ie, the face, hands, arms), cosmetic outcomes are important to patients and may increase satisfaction and compliance with the procedure.^93^, ^94^, ^95^

At-home topical therapies are widely used but may have poor compliance and variable adherence.^20^ This has been discussed in several studies, 1 discovered that those patients that were nonadherent were significantly less informed about how to correctly use the topical agent, including application time and frequency.^96^ There are also unpredictable reactions and inconsistent protocols for managing the local skin irritation induced by the mechanisms of the treatment. As seen routinely in medicine, the often-busy routine in outpatient clinics may not allow sufficient time for patient education to set up patients for success.^51^^,^^91^ This is compounded by the potential for patient confusion and hesitance to ask for clarification, especially in older patients. It has been suggested that sufficient pretreatment consultation may remove this obstacle and increase adherence.^91^ PDT removes many of the shortcomings of at-home compliance as it is performed in-office, does not require patient application, and allows clinical staff to complete treatment and postprocedure education. In a patient perception study, patients were significantly more satisfied with PDT vs 5-FU (*P* = .058) and preferred PDT to 5-FU (*P* < .001) or imiquimod (*P* = .031).^21^

Since AKs are a potential cancer development and progression marker, they must be treated in a timely and effective manner. PDT, in combination with other treatments, including topical or laser therapies, can be considered. There was universal agreement that sequential treatment and/or pretreatment with 5-FU, imiquimod, ablative lasers, microneedling, or heat application may yield better results than PDT alone. A study showed that use of sequential treatment of 5% imiquimod and aminolevulinic acid (ALA)-PDT (20%) on patients with at least 10 AKs on the face yielded significantly better results than ALA-PDT alone. At month 12, patients with sequential treatment had a 89.9% median reduction in AK lesions, while those who underwent ALA-PDT alone only had a median reduction of 74.5% (*P* = .0023).^64^^,^^97^ Similarly, other studies have confirmed similar trends with other pretreatments such as 5-FU. A study demonstrated that combined treatment with 5-FU and methyl aminolevulinate-PDT on AKs showed 75% clearance rates at 3 months while methyl aminolevulinate-PDT treatment alone only yielded 45% clearance.^63^^,^^64^

Currently, ALA formulations are the only United States Food and Drug Administration approved PDT photosensitizers remaining on the market. The 10% nanoemulsion gel is a newer formulation and has advantages over the original 20% ALA solution, such as enhanced epidermal penetration and protoporphyrin IX formulation, ease of application, and fewer local skin reactions during or after illumination.^67^, ^68^, ^69^ Our panelists agree that the use of the nanoemulsion gel has benefits, however, both formulations are effective at clearing AK lesions with their approved activating light sources.^69^ Although several protocols have set an optimal incubation time, the panelists agreed that other factors may also impact the determination of incubation time, including patient tolerance for pain, available time commitment, pre-treatment curettage of hyperkeratotic AKs, and number, extent and location of evident lesions.^98^, ^99^, ^100^ To combat these factors, the panelists suggested that shorter incubation times can be achieved with the use of other methods to enhance outcomes, such as occlusion, higher room temperature, heat application, curettage, and extra treatment sessions.^52^^,^^55^ However, caution must be taken as enhancing efficacy with these methods may induce more pain and additional tolerability or skin reactions, so clinicians should exercise clinical judgment.

As for light source, our panelists agreed that red light penetrates deeper and may be better for chemoprevention, however, both red and blue light are effective for PDT, especially AK treatment.^70^ Clinicians can use existing equipment they already own, whether red or blue light, for effective PDT treatment, and that specific new equipment is unnecessary. However, when selecting new equipment, red light may be preferred as it offers potential deeper treatment penetration.^70^ The simultaneous application and illumination painless PDT protocol was developed to reduce pain levels and increase patient tolerability and comfort.^74^ This involved application of 20% ALA and immediate illumination with blue light for 30 minutes.^74^ This bilaterally controlled, interpatient clinical trial demonstrated that all patients experienced significantly less pain undergoing painless PDT than conventional PDT, with mean visual analog scale pain scores of 0.52 vs 3.57.^74^ Panelists recommended that painless PDT can be effectively used to treat AKs, however at least 2 treatment sessions should be performed.^74^^,^^75^ Panelists also agreed that the addition of oral vitamin D3 may be beneficial during the simultaneous application and illumination painless PDT protocol. This was reflected in a study that showed the addition of vitamin D3 increased response rates from 40% to 70%.^75^ Other than the simultaneous application and illumination painless PDT protocol, alternative methods have been suggested in literature to mitigate pain.^101^, ^102^, ^103^, ^104^, ^105^, ^106^ This includes shortening photosensitizer incubation time to 15 minutes, taking short pauses during illumination, using lower fluence rates, and the use of fans, water misting, or cold air during PDT.^102^, ^103^, ^104^^,^^106^ All of these approaches have been implemented to reduce pain levels without negatively impacting AK lesion reduction.^101^, ^102^, ^103^, ^104^, ^105^, ^106^

Daylight PDT is another protocol involving application of photosensitizer in-office and utilizing daylight as the light source, despite being off label in the US market. Multiple European studies have reported that the use of daylight PDT can reduce discomfort and reduce local inflammatory reactions compared to conventional PDT, while maintaining similar efficacy.^71^ Several studies have investigated the efficacy of daylight PDT compared to conventional PDT. In one 12-week, randomized, controlled clinical trial investigating daylight PDT compared to conventional PDT in treating multiple mild and/or moderate facial or scalp AKs, it was found that daylight PDT was noninferior to conventional PDT, regardless of weather conditions.^107^ It was also found that daylight PDT was almost painless compared to conventional PDT (*P* < .001).^107^ The panelists agree that daylight PDT has been reported to be effective in some geographic locations and providers have had success using it.^71^^,^^72^ Daylight PDT can be considered an alternative for clinicians looking to incorporate PDT but do not have equipment or ability to acquire equipment to do so. Reimbursement and coding issues are still an obstacle for the time being.

Although the panelists agreed that field-directed PDT yields favorable outcomes for both nontransplant and OTRs, anecdotal evidence suggests that OTRs may not induce the same degree of inflammatory response and thus clearance rates with all field-directed therapies, not just PDT, and is likely due to chronic immunosuppression. The anecdotal evidence also suggested that to combat this, patients require longer treatment durations, need combinational therapies, or use longer PDT incubation times to get the same level of treatment response and post-treatment clearance.

## Conclusion

Through this modified Delphi method, we were able to anonymously harness opinions of 10 experts in the development of recommendations for the use of PDT in AKs. These are intended to help bridge gaps in the existing guidelines and provide further guidance for clinicians to improve cost-effective treatment approaches for a growing public health epidemic. This panel expects these, like any treatment guideline, will continue to evolve with the changing treatment landscape and growing clinical experience and evidence.

## Data availability

The data that support the findings of this study are available from the corresponding author upon reasonable request.

## Conflicts of interest

Dr Patel is a consultant for Regeneron, Almirall, Biofrontera, and Sun Pharmaceuticals; he serves on the speaker bureau for Regeneron and Sun Pharma. Dr Arron is a speaker and consultant for Regeneron and Castle Biosciences, a consultant for Replimune, an investigator and consultant for Enspectra Health, and an investigator for Sol-Gel/Premier Research. Dr Berman is a consultant and investigator for Sun Pharmaceuticals and Biofrontera. Dr Chapman is a clinical investigator for Sun Pharmaceutical and Biofrontera. Dr Jambusaria-Pahlajani is a consultant for Regeneron. Dr Martin is a consultant for Bristol Myers Squibb, DUSA/SUN, AbbVie, Ortho/Bausch Health, Galderma, Pfizer, LEO, Celgene, UCB, Trevi, Almirall, Lilly, Evelo, Organogenesis, and Janssen. He is also a speaker for InCyte, Dermavant, and Bristol Myers Squibb and on the advisory board for Bristol Myers Squibb, DUSA/SUN, AbbVie, Ortho/Bausch Health, Galderma, Pfizer, LEO, Celgene, Janssen, Horizon, UCB, Trevi, Almirall, Evelo Organogenesis, Novartis, Dermavant, InCyte, and Mindera. Dr Rossi is a consultant for Evolve CME, Almirall, Merz, Dynamed, Canfield Scientific, Allergan Inc, Evolus, Biofrontera, Quantia MD, Lam Therapeutics, Regeneron, and Cutera. He is also on the advisory board at Allergan Inc and Skinfix and the founder of DAR companies. Dr Schlesinger is an advisor, consultant, and investigator for Abbvie, Allergan (an Abbvie Company), Almirall, Biofrontera, Bristol Myers Squibb, Castle Biosciences, Galderma, Janssen, Lilly, L’Oreal, Pfizer, Regeneron, SUN Pharma, Verrica (consultant and speaker), and UCB. Dr Zeitouni is a consultant and investigator for Biofrontera. Dr Bhatia is an advisor, consultant, and investigator for Almirall, Biofrontera, Galderma, Leo, Ortho, Regeneron, Sun Pharmaceuticals, and Verrica.
